# Redox state and the sirtuin deacetylases are major factors that regulate the acetylation status of the stress protein NQO1

**DOI:** 10.3389/fphar.2022.1015642

**Published:** 2022-11-02

**Authors:** David Siegel, Peter S. Harris, Cole R. Michel, Rafael de Cabo, Kristofer S. Fritz, David Ross

**Affiliations:** ^1^ Department of Pharmaceutical Sciences, Skaggs School of Pharmacy and Pharmaceutical Sciences, University of Colorado Anschutz Medical Campus, Aurora, CO, United States; ^2^ Mass Spectrometry Facility, Skaggs School of Pharmacy and Pharmaceutical Sciences, University of Colorado Anschutz Medical Campus, Aurora, CO, United States; ^3^ Experimental Gerontology Section, Translational Gerontology Branch, National Institute on Aging, Baltimore, MD, United States

**Keywords:** NQO1, SIRTUIN, NADH, redox proteomics, acetylation, glutathione, SIRT2

## Abstract

The stress induced protein NQO1 can participate in a wide range of biological pathways which are dependent upon the interaction of NQO1 with protein targets. Many of the protein-protein interactions involving NQO1 have been shown to be regulated by the pyridine nucleotide redox balance. NQO1 can modify its conformation as a result of redox changes in pyridine nucleotides and sites on the C-terminal and helix seven regions of NQO1 have been identified as potential areas that may be involved in redox-dependent protein-protein interactions. Since post-translational modifications can modify the functionality of proteins, we examined whether redox-dependent conformational changes induced in NQO1 would alter lysine acetylation. Recombinant NQO1 was incubated with and without NADH then acetylated non-enzymatically by acetic anhydride or S-acetylglutathione (Ac-GSH). NQO1 acetylation was determined by immunoblot and site-specific lysine acetylation was quantified by mass spectrometry (MS). NQO1 was readily acetylated by acetic anhydride and Ac-GSH. Interestingly, despite a large number of lysine residues (9%) in NQO1 only a small subset of lysines were acetylated and the majority of these were located in or near the functional C-terminal or helix seven regions. Reduction of NQO1 by NADH prior to acetylation resulted in almost complete protection of NQO1 from lysine acetylation as confirmed by immunoblot analysis and MS. Lysines located within the redox-active C-terminus and helix seven regions were readily acetylated when NQO1 was in an oxidized conformation but were protected from acetylation when NQO1 was in the reduced conformation. To investigate regulatory mechanisms of enzymatic deacetylation, NQO1 was acetylated by Ac-GSH then exposed to purified sirtuins (SIRT 1-3) or histone deacetylase 6 (HDAC6). NQO1 could be deacetylated by all sirtuin isoforms and quantitative MS analysis performed using SIRT2 revealed very robust deacetylation of NQO1, specifically at K^262^ and K^271^ in the C-terminal region. No deacetylation of NQO1 by HDAC6 was detected. These data demonstrate that the same subset of key lysine residues in the C-terminal and helix seven regions of NQO1 undergo redox dependent acetylation and are regulated by sirtuin-mediated deacetylation.

## Introduction

NAD(P)H:quinone oxidoreductase 1 (NQO1) is a multifunctional protein and its many roles have been summarized in recent reviews (Lee et al., 2021; Ross and Siegel, 2021). Some of the diverse roles of NQO1, in addition to reduction of endogenous and xenobiotic quinones, include serving as a gatekeeper of the proteasome (Asher et al., 2005), regulation of microtubule acetylation (Siegel et al., 2021) and binding and regulation of mRNA translation (Castello et al., 2012; Di Francesco et al., 2016). These functions are dependent upon protein interactions between NQO1 and target molecules and have been shown to be redox-dependent (Tsvetkov et al., 2010; Salido et al., 2022). NQO1 is a flavin adenine dinucleotide (FAD)-containing homodimeric protein that can utilize reduced pyridine nucleotides (NADH or NADPH) to catalyze the two-electron reduction of substrates. The FAD cofactor in NQO1 is non-covalently bound and acts as a critical intermediary for hydride transfer between NA(P)H and substrate assisted by a tyrosine histidine relay (Li et al., 1995). On reduction, NQO1 undergoes a conformational change to accommodate and stabilize the reduced flavin (FADH_2_) cofactor (Faig et al., 2000). The ability of NQO1 to structurally adapt to NAD(P)H, and its propensity to interact with a wide range of other proteins, suggests that NQO1 may serve as a redox sensor that can alter its conformation in response to changes in pyridine nucleotide concentrations (Ross and Siegel, 2017; Siegel et al., 2018).

While the specific regions on NQO1 that participate in redox-dependent protein-protein interactions have not been determined recent studies have identified two sites as potential candidates. After reduction by NADPH the C-terminal and helix seven regions of NQO1 are no longer immunoreactive to antibodies that target epitopes located within these regions (Siegel et al., 2018). In addition, reduction of NQO1 by NADPH also prevented proteolytic cleavage of the C-terminus (Chen et al., 1994). Further strengthening the case for the C-terminal and helix seven regions as important sites for redox-dependent protein-protein interactions are the large number of post-translational modifications (PTMs) which have been mapped to these regions. PTMs regulate many aspects of protein function including catalytic activity, protein-protein interactions, subcellular localization and ultimately may serve as signals for protein degradation (Karve and Cheema, 2011). Even relatively minor chemical modifications such as lysine acetylation have been shown to dramatically alter the functionality of proteins (Narita et al., 2019). NQO1 is well suited as a target protein for lysine acetylation since 9% of the amino acids (48/548) in the homodimeric protein are lysine amino acids many of which are located within or near the redox active C-terminal or helix seven regions (Li et al., 1995).

In this study, we utilized acetic anhydride, a physiologically relevant metabolite S-acetylglutathione (Ac-GSH) and the sirtuin deacetylases to examine the dynamic acetylation of oxidized and reduced forms of NQO1. Our novel approach examined how changes in pyridine-nucleotide redox balance affect the structure and acetylation status of NQO1. Quantitative MS analysis was performed using acetylated NQO1 and SIRT2 to identify target lysines in key structural regions in NQO1.

## Materials and methods

### Reagents

Acetic anhydride, NAD^+^, NADH, FAD, 2,6-dichlorophenolindophenol (DCPIP), bovine serum albumin (BSA), L-glutathione (reduced, GSH) and dimethyl sulfoxide (DMSO) were obtained from Millipore-Sigma (Burlington, MA). S-acetyl-L-glutathione (Ac-GSH) was purchased from BOC Sciences (Shirley, NY). The SIRT2 inhibitor AGK2 was purchased from Santa Cruz Biotechnology (Dallas, TX) and dissolved in sterile DMSO.

### Purified proteins and antibodies

Purified recombinant human NQO1 (rhNQO1,DT-diaphorase, #D1315) was purchased from Millipore-Sigma as a lyophilized powdered and reconstituted with 500 µL of _dd_H_2_O followed by 5 µM FAD. The protein was aliquoted and stored at -80°C. NQO1 demonstrated >98% purity as determined by SDS-PAGE (Supplementary Figure S1). Recombinant human SIRT1 (#10011190), recombinant human SIRT2 (#10011191) and recombinant human SIRT3 (#10011194) were purchased from Cayman Chemical (Ann Arbor, MI). SIRT1 and SIRT3 were used undiluted from the manufacture while SIRT2 was diluted in 50 mM sodium phosphate buffer (pH 7.2), containing 100 mM sodium chloride and 20% (v/v) glycerol. Recombinant human HDAC6 (#BML-SE508-0050) was purchased from Enzo Life Sciences (Farmingdale, NY). Rabbit anti-acetylated-lysine antibody (#9441) was purchased from Cell Signaling Technology (Danvers, MA) and used at a dilution of 1:800. HRP-conjugated goat anti-rabbit IgG was purchased from Jackson Immuno Research Labs (West Grove, PA).

### Acetylation of NQO1 by acetic anhydride

Stock solution of acetic anhydride was prepared in methanol immediately prior to addition. Reactions were performed in 200 mM potassium phosphate with 3.2 µM NQO1 in the absence or presence of 500 µM NADH or NAD^+^. Reactions were started by the addition of 125 µM acetic anhydride and were allowed to react for 5min at 25°C then 10 µL of the reaction was removed and added to 90 µL Laemmli SDS-sample buffer (2X reducing). Samples (20 µL) were then analyzed for lysine acetylation by immunoblot analysis.

### Acetylation of NQO1 by Ac-GSH

Stock solution of Ac-GSH was prepared in _dd_H_2_O immediately before addition. Reactions (20 µL) were performed in 100 mM potassium phosphate buffer (pH 7.8) with 3.2 µM NQO1 in the absence and presence of 500 µM NADH. Reactions were started by the addition of 5 mM Ac-GSH and were allowed to react for 1 h at 37°C then terminated by the addition of 6 µL Laemmli SDS-sample buffer (6X reducing, Boston BioProducts, Milford, MA). Samples (20 µL) were then analyzed for lysine acetylation by immunoblot analysis.

### NQO1 catalytic activity

Reactions were performed in 200 mM potassium phosphate with 3.2 µM NQO1 in the absence or presence of 200 µM NADH. Reactions were started by the addition of 125 µM acetic anhydride and were allowed to react for 5min at 25°C after which 10 µL of the reaction was removed and diluted 50X in ice-cold stop buffer (50 mM Tris-HCl (pH 7.4) containing 5 mg/ml BSA and 5 µM FAD). To measure NQO1 catalytic activity a 5 µL aliquot of the diluted reaction was added to a cuvette with buffer (25 mM Tris-HCl, pH 7.4 containing 1 mg/ml BSA and 5 µM FAD), 200 µM NADH and 40 µM DCPIP (1 ml total volume) and the decrease in absorbance at 600 nm was measured over 1 min at 25°C using a HP8452 diode array spectrophotometer (Benson et al., 1980).

### Deacetylation of Ac-NQO1

For studies with deacetylase enzymes NQO1 was acetylated by Ac-GSH then washed free of unreacted Ac-GSH. Briefly, samples (2 × 240 µL) each containing 3.2 µM NQO1 and 5 mM Ac-GSH in 100 mM potassium phosphate buffer (pH 7.8) were allowed to react for 1 h at 37°C. Samples were then place on ice to stop the acetylation reaction then loaded onto pre-washed ultra-concentration units (Microcon Ultracel YM-30, 30 kDa cutoff, Millipore-Sigma) and centrifuged at 13K for 10min at 4°C. The retained protein was washed with 200 µL of 25 mM Tris-HCl (pH 7.4) containing 5 µM FAD then recentrifuged at 13K for 10min at 4°C. The above wash procedure was repeated (2 washes total) and Ac-NQO1 was collected and diluted to 2 µg/µL with 25 mM Tris-HCl (pH 7.4) containing 5 µM FAD. Ac-NQO1 was stored at -80°C. Deacetylase reactions (20 µL) were performed in 50 mM Tris HCl, pH 7.4, 137 mM NaCl, 2.7mM KCl, 1 mM MgCl_2_ (1X SIRT1 direct assay buffer, #10010993, Cayman Chemical) with 3.2 µM Ac-NQO1 at 37°C for the indicated times. Reactions with SIRT1-3 were performed in the absence and presence of 1 mM NAD^+^. Reactions were terminated by the addition of 6 µL Laemmli SDS-sample buffer (6X reducing). Samples (20 µL) were then analyzed for lysine acetylation by immunoblot analysis.

### Immunoblotting

Samples were heated to 90°C for 10 min then centrifuged at 16,000 x g (Heraues Biofuge Pico) for 1 min. Samples were applied to a 12% precast minigel (Bio-Rad Laboratories, Hercules CA) then electrophoresed in 25 mM Tris (base), 192 mM glycine and 0.1% SDS at room temperature. Protein(s) was transferred to a polyvinylidene difluoride (PVDF, 0.45 µM) membrane in 10 mM Tris (base), 192 mM glycine with 20% (v/v) methanol at 75 V for 1.5 h at 25°C. Following transfer membranes were blocked in 10 mM Tris-HCl, pH 7.8, 150 mM NaCl and 0.2% (v/v) Tween-20 (TBST) containing 5% (w/v) non-fat dry milk (blocking buffer) for 30 min at 25°C. For studies using Ac-GSH the transfer of NQO1 was visualized by incubating membranes in Ponceau S protein stain (Ponceau S Solution, Millipore Sigma) for 10 min at 25°C. Afterwards membranes were briefly rinsed with TBST and photographed. Membranes were then washed extensively in TBST to remove any remaining Ponceau S stain and primary antibodies diluted in blocking buffer (4 ml) were applied to membranes overnight at 4°C. After incubation with primary antibodies membranes were washed extensively in TBST followed by the addition of a horseradish peroxidase-conjugated secondary antibody diluted 1:4000 in blocking buffer (20 ml) for 30min at 25°C. Membranes were washed extensively with TBST and protein bands were visualized with enhanced chemiluminescence (GE Healthcare Life Sciences, Pittsburgh, PA) using a Bio-Rad Gel-Doc imager.

### Mass spectrometry

#### Acetylation of NQO1 by acetic anhydride

Reactions (20 µL) were performed in 200 mM potassium phosphate buffer (pH 7.4) with 2 µg NQO1 (3.2µM, 64 pmol) in the absence and presence of 200 µM NADH. Reactions were initiated by the addition of 125 µM acetic anhydride and were allowed to react for 5 min at 25°C then terminated by the addition of 6 µL of Laemmli SDS-sample buffer (6X reducing).

#### Acetylation of NQO1 by Ac-GSH

Reactions (20 µL) were performed in 100 mM potassium phosphate buffer (pH 7.4) with 2 µg NQO1 (3.2µM, 64 pmol) in the absence and presence of 500 µM NADH. Reactions were initiated by the addition of 5 mM Ac-GSH and were allowed to react for 1 h at 37°C then terminated by the addition of 6 µL of Laemmli SDS-sample buffer (6X reducing).

#### Deacetylation of NQO1

Reactions (20 µL) were performed in 1X SIRT1 direct assay buffer (above) with 2 µg Ac-NQO1 (3.2µM, 64 pmol) in the absence or presence of 100 ng of SIRT2 with 1 mM NAD^+^. Reactions were allowed to react for indicated times at 37°C then terminated by the addition of 6 µL of Laemmli SDS-sample buffer (6X reducing).

Solvents for in-gel trypsin digestion were of HPLC grade purity or greater. NQO1 acetylation/deacetylation was achieved as described above. Samples (20 µL) were applied to a 10% SDS-PAGE gel and electrophoresed approximately 10 mm into the resolving gel. Gels were then Coomassie stained for 1 h and destained in water overnight at 25°C. NQO1 protein bands were excised from the gel, and proteolysis was performed using a standard in-gel digestion procedure (Fritz et al., 2012). Excised protein bands were completely destained in 50% acetonitrile (ACN), 50% 50 mM ammonium bicarbonate (NH_4_HCO_3_), dehydrated in 100% ACN and then reduced with 10 mM dithiothreitol in 50 mM NH_4_HCO_3_ for 45 min at 60°C. The gel pieces were then treated with 40 mM iodoacetamide in 50 mM NH_4_HCO_3_ in the dark for 30 min at 25°C then washed twice in 50 mM NH_4_HCO_3_ and resuspended in 150 μL of 50 mM NH_4_HCO_3_ with 0.9 μg porcine-modified trypsin (cat #V5111, Promega, Madison, WI), and digested overnight at 37°C. Samples were then dried in a SpeedVac and resuspended in 40 ul of 0.1% formic acid, 3% ACN in water. Acetylated peptides were quantified by mass spectrometry (Agilent 6550) using an accurate mass and retention time (AMRT) library approach, as previously described (Ali et al., 2020). A more detailed description of the methods used in the mass spectrometric analysis are provided in the Supplementary Material. To determine statistical significance between treatments (n = 4 per treatment) a moderated *t*-test was performed with Benjamini-Hochberg multiple testing corrections in those experiments with two sample groups and an ANOVA was performed with Benjamini-Hochberg multiple testing corrections in the experiment with three groups. Statistically significant acetyl lysine sites reported are those sites that had a corrected *p*-value of less than 0.05 between groups.

## Results

**FIGURE 1:**
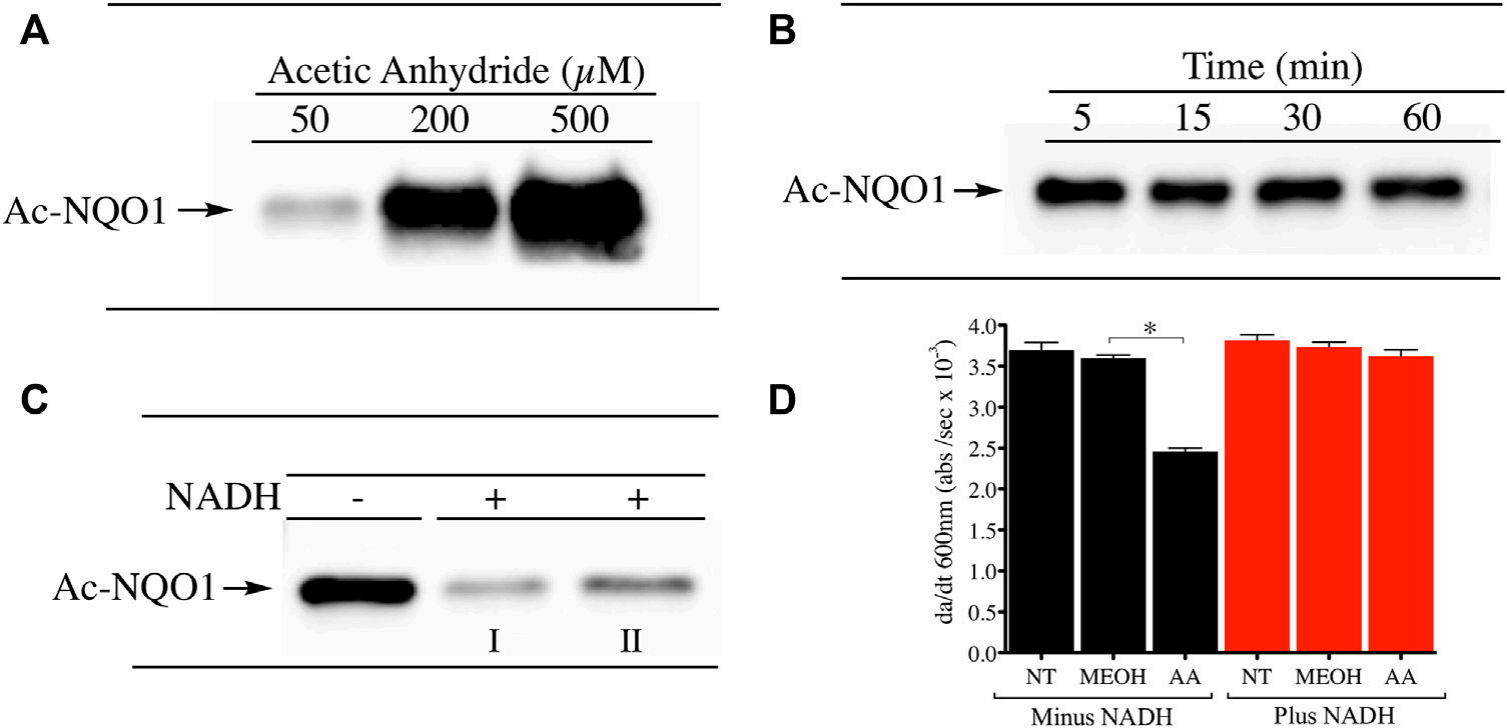
Acetylation of NQO1 by acetic anhydride. Acetic anhydride dependent lysine acetylation of NQO1 was monitored by immunoblot analysis using anti-acetylated lysine antibodies **(A)** NQO1was treated with acetic anhydride (50–500 µM) for 1 h **(B)** NQO1was incubated with acetic anhydride (125 µM) for the indicated times **(C)** NQO1 was incubated in the absence and presence of NADH (500 µM) then treated with acetic anhydride (125 µM) for 5 min. Reactions performed in the presence of NADH were carried out in duplicate (I, II) **(D)** NQO1 catalytic activity was measured in reactions containing NQO1 in the absence and presence of NADH then treated with either methanol (solvent control) or acetic anhydride for 5 min. Results are shown as the mean ± SD of three independent determinations. NT, no treatment; MEOH, methanol; AA, acetic anhydride. *p <0.05 unpaired *t*-test. Reaction conditions are provided in the Materials and methods section.

The conformational change induced in NQO1 following reduction by NAD(P)H is a regulatory mechanism by which redox dependent post-translational modifications (PTMs) can impact its structure and function. To investigate whether reduction by NADH can alter the PTM landscape of NQO1 we exposed purified rhNQO1 to the acetylating reagent acetic anhydride and monitored lysine acetylation using immunoblot analysis with a pan anti-acetylated lysine antibody. Treatment of NQO1 with acetic anhydride resulted in concentration-dependent lysine acetylation on NQO1 (Figure 1A). Lysine acetylation generated by acetic anhydride was very rapid with peak levels detected after only 5 min of exposure (Figure 1B). These data confirmed that in the oxidized conformation lysine residues on NQO1 can easily be acetylated through chemical means. In contrast, pretreatment of NQO1 with NADH before acetic anhydride treatment resulted in near complete protection against lysine acetylation (Figure 1C). Spectrophotometric analysis (260–450 nm) of NADH in the presence and absence of acetic anhydride at pH 7.4 (25°C) were identical indicating that acetic anhydride itself does not directly oxidize NADH under these conditions (Supplementary Figure S2). To determine whether acetylation of NQO1 by acetic anhydride could affect the catalytic activity of NQO1 we treated NQO1 with acetic anhydride (using the same conditions shown in Figure 1C) then measured NQO1 catalytic activity spectrophotometrically using the reduction of DCPIP. In these studies, acetylation of NQO1 by acetic anhydride resulted in a 37% decrease in NQO1 activity (Figure 1D). However, if NQO1was treated with NADH prior to acetic anhydride no loss of catalytic activity of NQO1 could be detected. These data support the immunoblot results and the hypothesis that reduction of NQO1 by NADH alters the conformation of NQO1 which prevents lysine acetylation. The acetylation of lysine residues in the C-terminus of NQO1 may also be responsible for the moderate decrease in catalytic activity since basic amino acids in this region have been hypothesized to bind with the positively charged pyrophosphate in NAD(P)H when NQO1 is in the oxidized conformation (Zhao et al., 1997).

**FIGURE 2:**
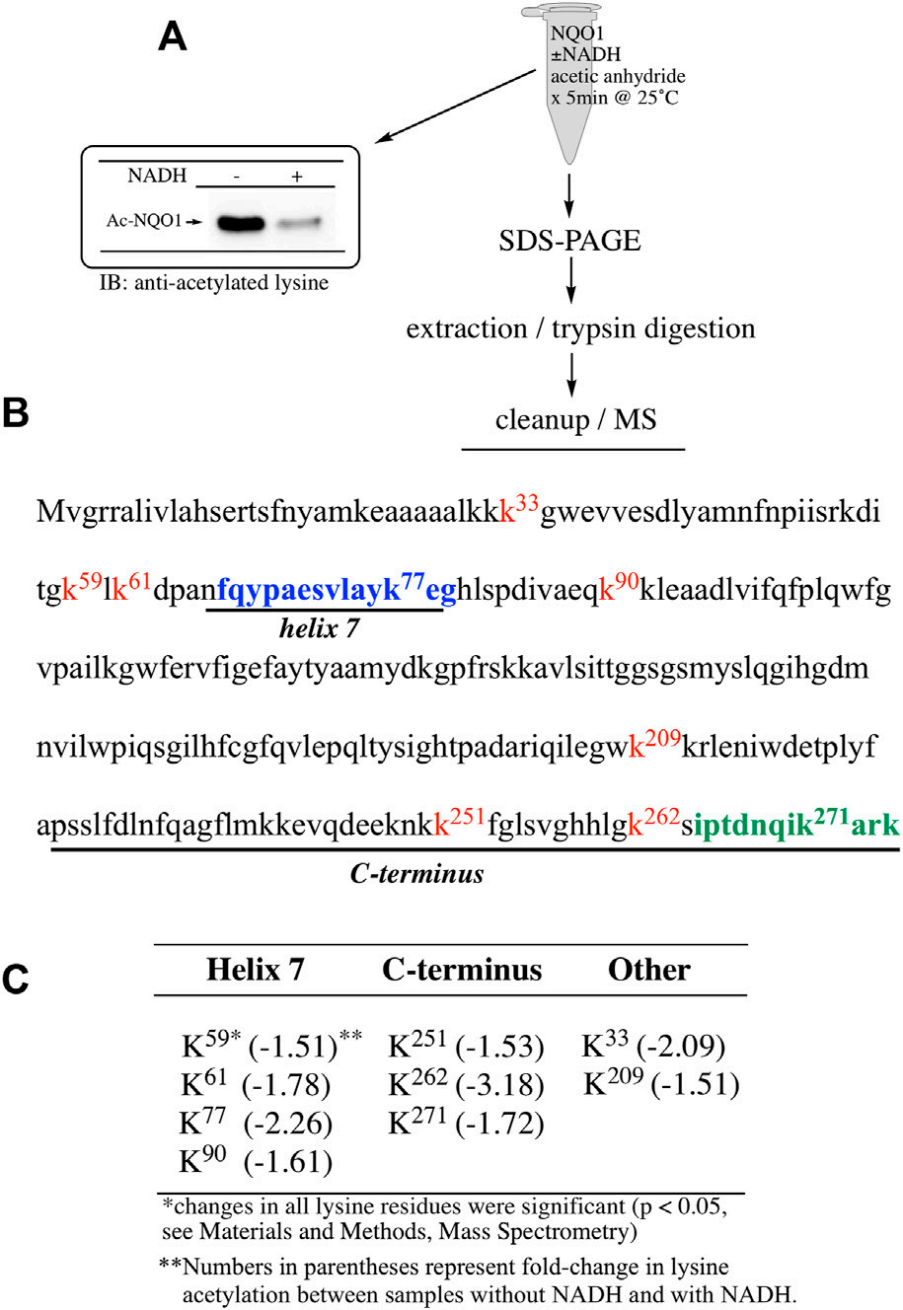
Redox dependent acetylation of NQO1. Quantitative MS analysis was used to identify acetylated lysine residues following treatment with acetic anhydride in oxidized (-NADH) and reduced (+NADH) forms of NQO1 **(A)** For MS analysis NQO1(2 µg) was incubated in the absence and presence of NADH (500 µM) then treated with acetic anhydride (125 µM) for 5 min at 25°C. Samples (*n* = 4 per treatment) were then prepared for MS as shown above. Representative immunoblot showing the levels of lysine acetylation on NQO1 using the reaction conditions described above **(B)** Diagram showing the amino acid sequence of hNQO1 and the identity of lysine residues (k^x^ red text) which were discovered to be acetylated by acetic anhydride. The location of redox-dependent regions of NQO1 (helix seven and C-terminus) identified in previous studies using redox-dependent anti-NQO1 antibodies (A180 epitope in blue text, C-term epitope in green text) **(C)** The majority (7 of 9) of the lysine residues which demonstrated a decrease in acetylation with NADH lie within or very near the redox active helix seven and C-terminus regions. All nine lysine residues that were found to be acetylated by acetic anhydride showed a significant decrease (*p* <0.05) in acetylation when NQO1 was pretreated with NADH.

To determine which specific lysine residues on NQO1 were acetylated by acetic anhydride and which lysine residues showed redox-dependent acetylation we performed quantitative MS acetylomics analysis on NQO1 treated with acetic anhydride in the absence and presence of NADH. Results from this analysis are shown in Figure 2. Treatment of NQO1 with acetic anhydride in the absence of NADH resulted in the detection of nine acetylated lysine residues (Figure 2B). Moreover, in samples pretreated with NADH we observed a significant reduction in the levels of acetylation of these same nine lysine residues (Figure 2C). Interestingly, the location of seven of the nine lysine residues on NQO1 which were discovered to be acetylated are located in or near the redox-active C-terminal or helix seven regions in NQO1 (Figures 2B,C).

**FIGURE 3:**
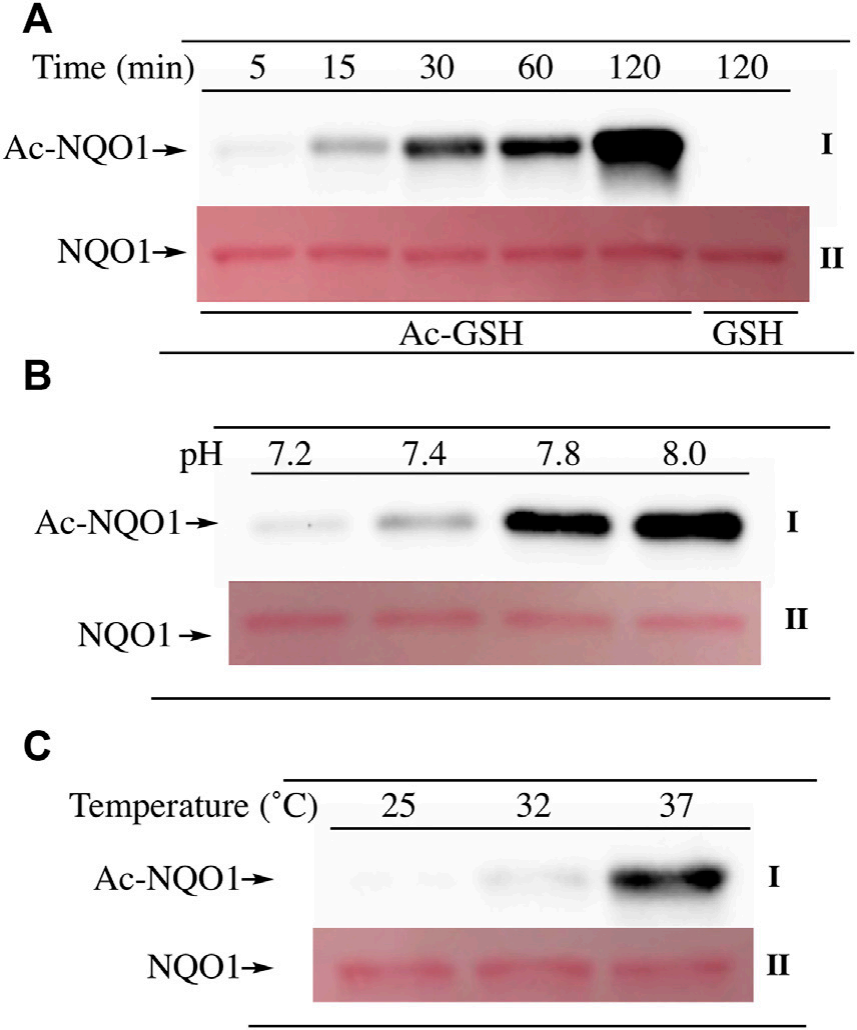
Acetylation of NQO1 by Ac-GSH. Ac-GSH dependent lysine acetylation of NQO1 was monitored by immunoblot analysis using anti-acetylated lysine antibodies **(I) (A)** NQO1 was incubated with Ac-GSH for the indicated times **(B)** NQO1 was incubated with Ac-GSH for 1 h at the indicated pH values **(C)** NQO1 was incubated with Ac-GSH for 1 h at 25°C, 32°C and 37°C. Ponceau S. staining was used to monitor protein loading and transfer (II). GSH was utilized as a non-acetylating control. Reaction conditions are provided in the Materials and methods section.

**FIGURE 4:**
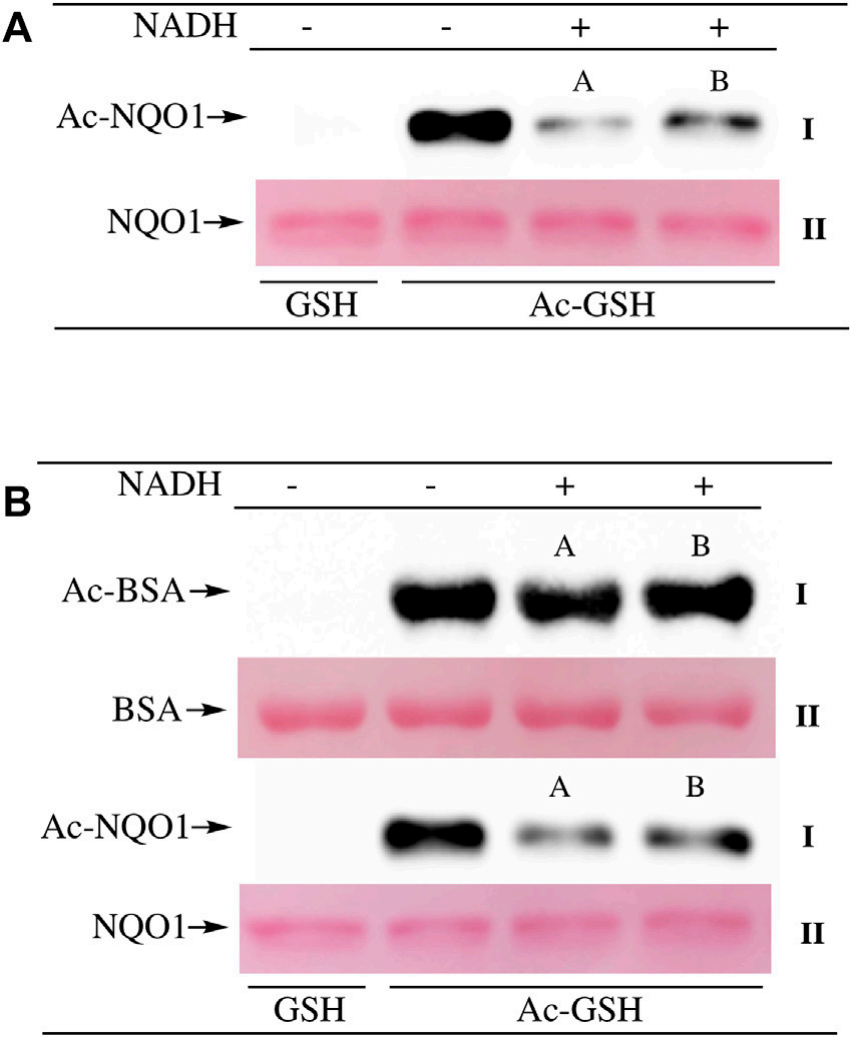
NADH-mediated reduction of NQO1 protects against lysine acetylation. Ac-GSH dependent lysine acetylation of NQO1 was monitored by immunoblot analysis using anti-acetylated lysine antibodies **(I) (A)** NQO1 was incubated with Ac-GSH in the absence and presence of NADH for 1 h. Reactions in the presence of NADH were performed in duplicate **(A,B) (B)** NQO1(2 µg) and BSA (2 µg) were incubated with Ac-GSH in the absence and presence of NADH for 1 h at 37°C. Reactions in the presence of NADH were performed in duplicate **(A,B)**. Ponceau S. staining was used to monitor protein loading and transfer (II). GSH was utilized as a non-acetylating control. Reaction conditions are provided in the Materials and methods section.

**FIGURE 5:**
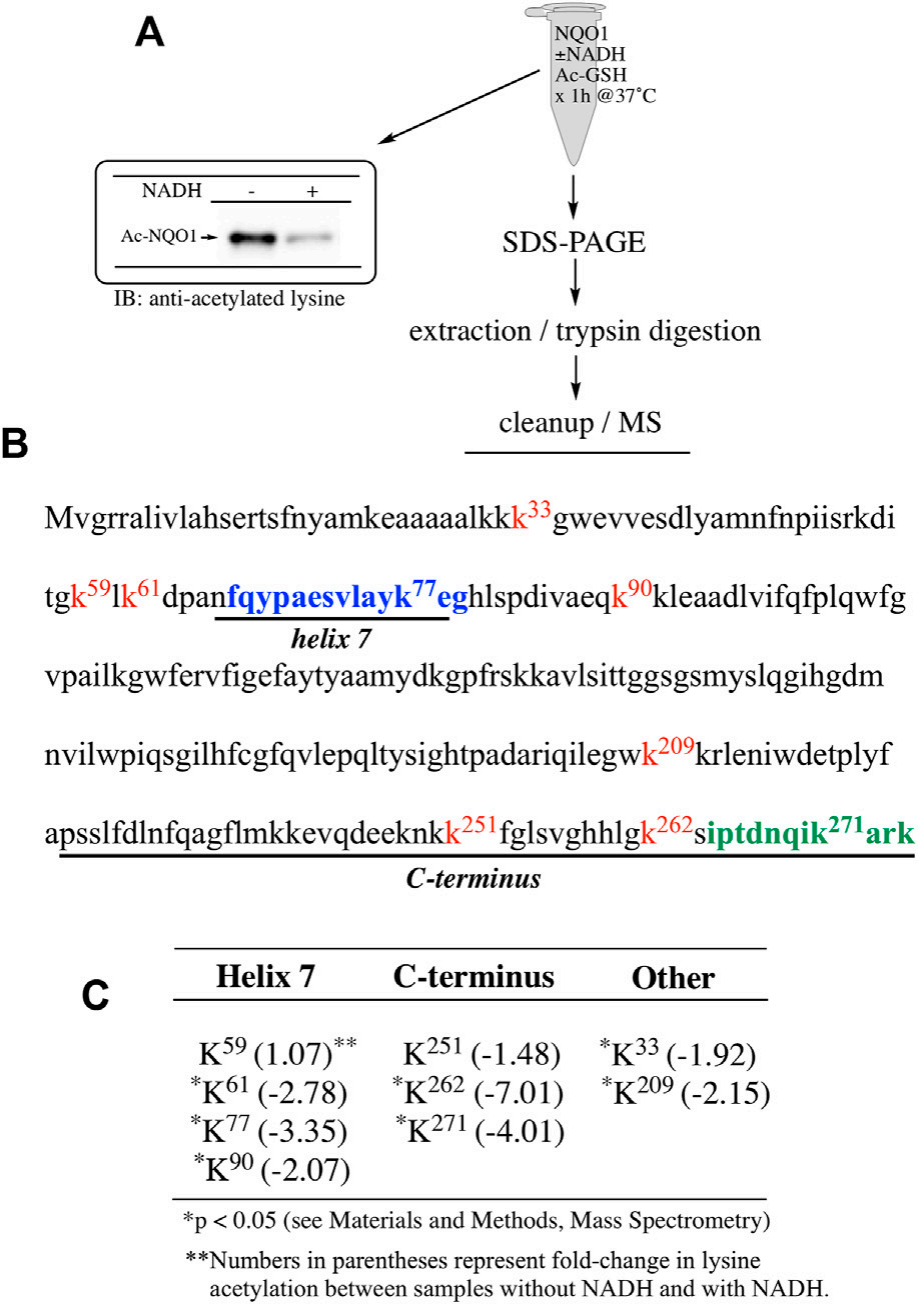
Redox dependent acetylation of NQO1 by Ac-GSH. Quantitative MS analysis was used to identify acetylated lysine residues following treatment with Ac-GSH in oxidized (-NADH) and reduced (+NADH) forms of NQO1 **(A)** For MS analysis NQO1(2 µg) was incubated in the absence and presence of NADH (500 µM) then treated with Ac-GSH (5 mM) for 1 h at 37°C. Samples (*n* = 4 per treatment) were then prepared for MS as shown above. Representative immunoblot showing the levels of lysine acetylation on NQO1 using the reaction conditions described above **(B)** Diagram showing the amino acid sequence of hNQO1 and the identity of lysine residues (k^x^ red text) which were discovered to be acetylated by Ac-GSH. The location of redox-dependent regions of NQO1 (helix seven and C-terminus), identified in previous studies using redox-dependent anti-NQO1 antibodies (A180 epitope in blue text, C-term epitope in green text) **(C)** The majority (7 of 9) of the lysine residues which demonstrated a decrease in acetylation with NADH lie within or very near the redox active helix seven and C-terminus regions. *Denotes lysine residues which showed a significant decrease (*p* <0.05) in the levels of acetylation when NQO1 was pretreated with NADH.

To examine lysine acetylation of NQO1 under more physiological conditions we replaced acetic anhydride with S-acetylglutathione (Ac-GSH). Treatment of NQO1 with Ac-GSH resulted in time-dependent lysine acetylation of NQO1. In contrast to acetic anhydride, lysine acetylation induced by Ac-GSH was slower and continued to increase up to 2 h after addition (last time point measured, Figure 3A). No lysine acetylation on NQO1 was detected when GSH was used in place of Ac-GSH confirming the specificity of the reaction and immunoblot assay (Figure 3A). Acetylation of NQO1 by Ac-GSH was pH dependent with increasing levels of acetylation detected as the pH was increased (Figure 3B). Similar results have been observed with the non-enzymatic acetylation of proteins by acetyl coenzyme A (Wagner and Payne, 2013). Acetylation of NQO1 by Ac-GSH was temperature-dependent with only minor levels of lysine acetylation detected below 37°C (Figure 3C). These data demonstrate that Ac-GSH can directly acetylate lysine residues on NQO1 under physiological conditions. Similar to what was observed with acetic anhydride, pretreatment of NQO1 with NADH prior to exposure to Ac-GSH resulted in near complete protection against lysine acetylation (Figure 4A). While pretreatment with NADH did protect NQO1, NADH pretreatment did not protect BSA against lysine acetylation by Ac-GSH (Figure 4B). These data show that NADH provides a protective effect by inducing a specific conformational change in NQO1 and not by directly reacting with Ac-GSH. The identities of the lysine amino acids in NQO1 acetylated by Ac-GSH were determined by MS analysis. Similar to results observed with acetic anhydride, the same lysine residues on NQO1 were found to be acetylated by Ac-GSH and pretreatment of NQO1 with NADH prevented these lysine residues from being acetylated by Ac-GSH (Figures 5B,C).

**FIGURE 6:**
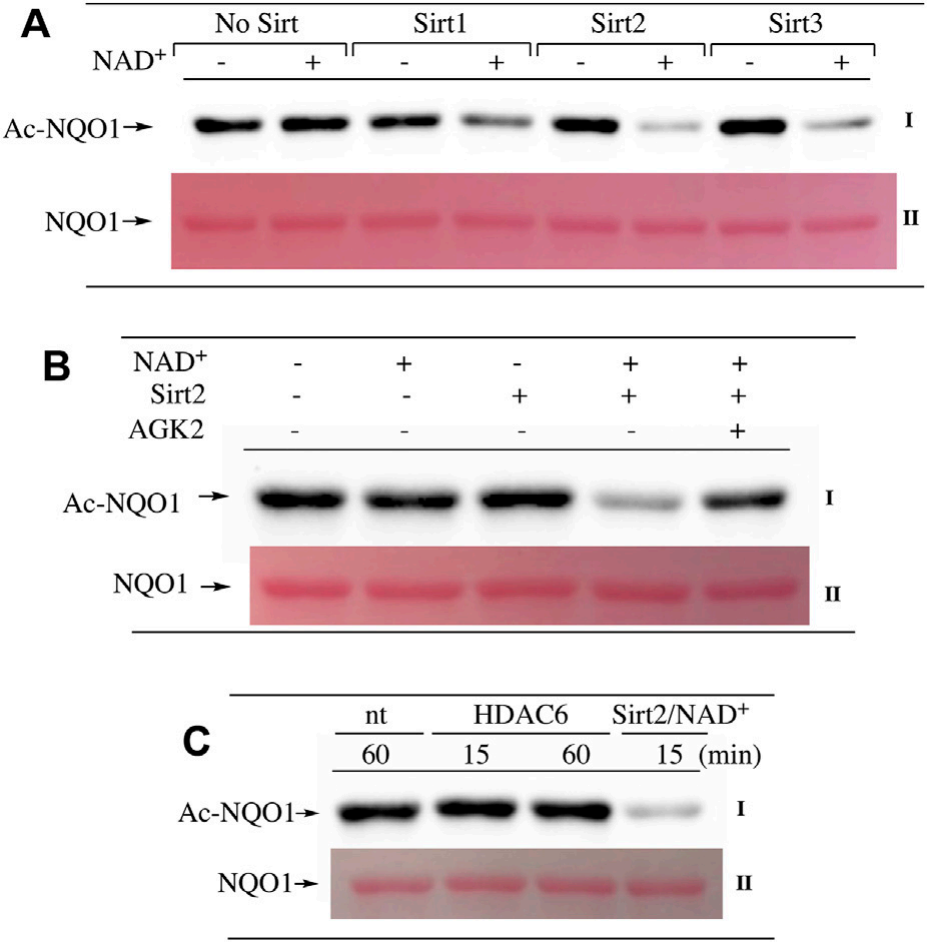
Deacetylation of NQO1. The deacetylation of NQO1 was monitored by immunoblot analysis using anti-acetylated lysine antibodies. Ac-NQO1 was generated by incubation of NQO1 with Ac-GSH as described in the Materials and methods section **(A)** Ac-NQO1 was incubated with purified SIRT1 (470 ng), SIRT2 (100 ng) or SIRT3 (830 ng) in the absence and presence of 1 mM NAD^+^ for 15 min **(B)** Ac-NQO1 was incubated with SIRT2 (100 ng) in the absence and presence of 1 mM NAD^+^ and the sirtuin inhibitor AGK2 (100 µM) for 15min **(C)** Ac-NQO1 was incubated with HDAC6 (1 µg) or SIRT2 (100 ng) and NAD^+^ (1 mM) for the indicated times. Reaction conditions are described in the Materials and methods section.

**FIGURE 7:**
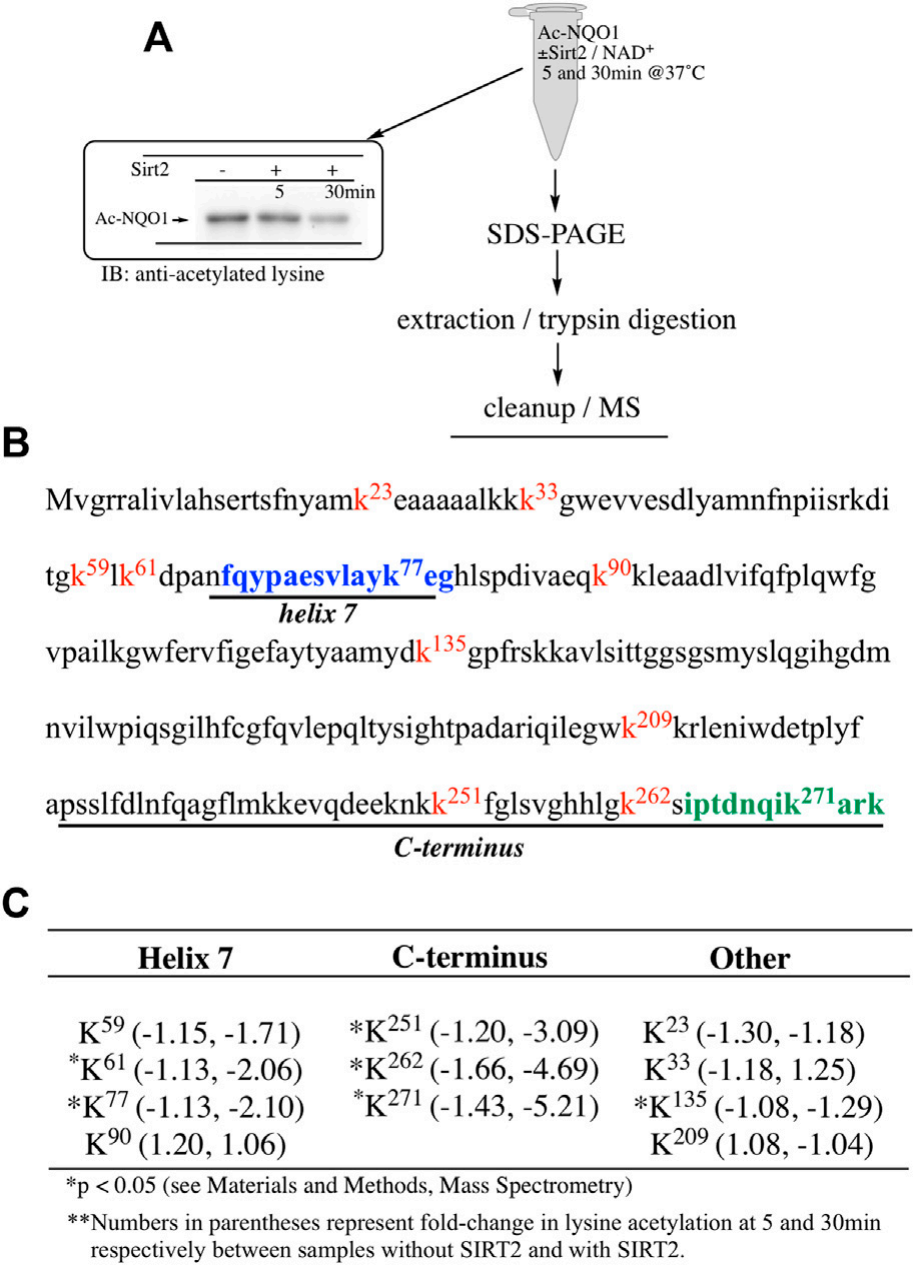
Sirt2-dependent deacetylation of NQO1. Quantitative MS analysis was used to identify lysine residues on NQO1 that were deacetylated by SIRT2/NAD^+^ **(A)** For MS analysis Ac-NQO1(2 µg) was incubated in the absence and presence of SIRT2 (100 ng) and NAD^+^ (1 mM) for 5 and 30 min at 37°C. Samples (*n* = 4 per treatment) were then prepared for MS as shown above. Representative immunoblot showing the levels of lysine acetylation on NQO1 using the reaction conditions described above **(B)** Diagram showing the amino acid sequence of hNQO1 and the identity of lysine residues (k^x^ red text) which were discovered to be acetylated by Ac-GSH. The location of redox-dependent regions of NQO1 (helix seven and C-terminus), identified in previous studies using redox-dependent anti-NQO1 antibodies (A180 epitope in blue text, C-term epitope in green text) **(C)** *Denotes lysine residues which showed a significant decrease (*p* <0.05) in the levels of acetylation when NQO1 was treated with SIRT2/NAD^+^.

Our ability to generate purified acetylated NQO1 enabled us to examine the role of deacetylase enzymes in the PTM landscape of NQO1. Acetylated NQO1 was prepared by treating purified NQO1 with Ac-GSH (5 mM × 1 h at 37°C) then washing the protein free of unreacted Ac-GSH. For deacetylation studies, Ac-NQO1 was exposed to SIRT1, SIRT2 or SIRT3 in the absence of presence of NAD^+^ (NAD^+^ is a sirtuin cofactor). Results from these studies clearly show that these sirtuin isoforms (SIRT1-3) can catalyze NAD^+^-dependent lysine deacetylation on NQO1 (Figure 6A). Deacetylation of Ac-NQO1 by SIRT2 was NAD^+^ dependent and inhibited by the sirtuin inhibitor AGK4 (Figure 6B). In these studies, HDAC6 did not catalyze the deacetylation of Ac-NQO1 under conditions where we observed SIRT2/NAD^+^- dependent deacetylation (Figure 5C). The purified HDAC6 used in these studies was confirmed to be catalytically active under these conditions (Supplementary Figure S3). MS analysis confirmed the immunoblot data and showed that the majority (7/11) of the lysine amino acids acetylated by Ac-GSH could be deacetylated by SIRT2/NAD^+^ (Figure 7B,C). K^262^ and K^271^ in the C-terminal region showed the greatest level of deacetylation at 5min suggesting that the C-terminal region may be the initial target for SIRT2.

## Discussion

Lysine acetylation is a ubiquitous PTM found on nearly all proteins which reflects cellular metabolism through Ac-CoA and Ac-GSH concentrations and can affect many protein functions including enzymatic activity, protein-protein interactions and stability (Narita et al., 2019). In these studies, we initially utilized direct chemical acetylation of the redox sensing protein NQO1 to characterize the acetylation landscape in oxidized and reduced conformations. Using mass spectrometry-based acetylomics analysis we identified lysine residues in NQO1 that were acetylated following treatment with either acetic anhydride or Ac-GSH. Our results demonstrate that despite a large number of lysine residues in NQO1 (48 lysine residues from a total of 548 amino acids; 9%) only a small subset were acetylated when NQO1 was in the oxidized conformation. Importantly, when NQO1 adopted the reduced conformation (plus NADH) these lysine residues were unable to be acetylated. The identity of the acetylated lysine amino acids in NQO1 are shown in Table 1 and they were predominantly located in the C-terminal and helix seven regions. Using both acetic anhydride- and Ac-GSH -mediated acetylation, the same lysine residues were acetylated and seven of nine acetylated lysine residues were located in the C- terminal and helix seven regions.

**TABLE 1:** Comparison of the NQO1 acetylome between chemically generated samples with purified NQO1 and biological samples from human cell lines obtained from PhosphoSite.

Acetic Anhydride	Ac-GSH	PhosphoSite (hNQO1)^a^
K^33^	K^33^	K^31^
K^59b^	K^59^	K^59^
K^61^	K^61^	K^61^
K^77^	K^77^	K^77^
K^90^	K^90^	K^90^
K^209^	K^209^	K^209^/K^210^
K^251c^	K^251^	K^251^
K^262^	K^262^	K^262^
K^271^	K^271^	

^a^ https://www.phosphosite.org/proteinAction.action?id=14721&showAllSites=true

^b^ Lysine residues in red are located in or near helix-7.

^c^ Lysine residues in green are located on the C-terminus.

Interestingly, the same subset of lysine residues in the C-terminal and helix seven regions of NQO1 have been identified as targets of acetylation in biological samples as shown on the PhosphoSite website (Table 1) which is a large data base of PTMs on human proteins (Hornbeck et al., 2015). The near exact match between our studies using non-enzymatic acetylation of purified NQO1 and biological data from human cell lines (PhosphoSite) indicates that the lysine residues we have identified have biological significance. Their location in or near helix-7 and the C-terminus, two regions of NQO1 which have previously been shown to undergo a redox-dependent change in conformation, suggests strongly that these lysine amino acids play a pivotal role in redox-dependent protein-protein interactions involving NQO1. Examples of interactions of NQO1 with other proteins have been reviewed (Ross and Siegel, 2021) and include p53/p73 (Asher et al., 2005), PGC1-α (Adamovich et al., 2013) and Hif1-α (Oh et al., 2016). Further strengthening this hypothesis is data from PhosphoSite that shows both the C-terminal and helix seven regions have large numbers of other PTMs, including ubiquitination, phosphorylation and sumoylation, indicating that these areas on NQO1 are in close association with many other proteins (https://www.phosphosite.org/proteinAction.action?id=14721&showAllSites=true).

**FIGURE 8:**
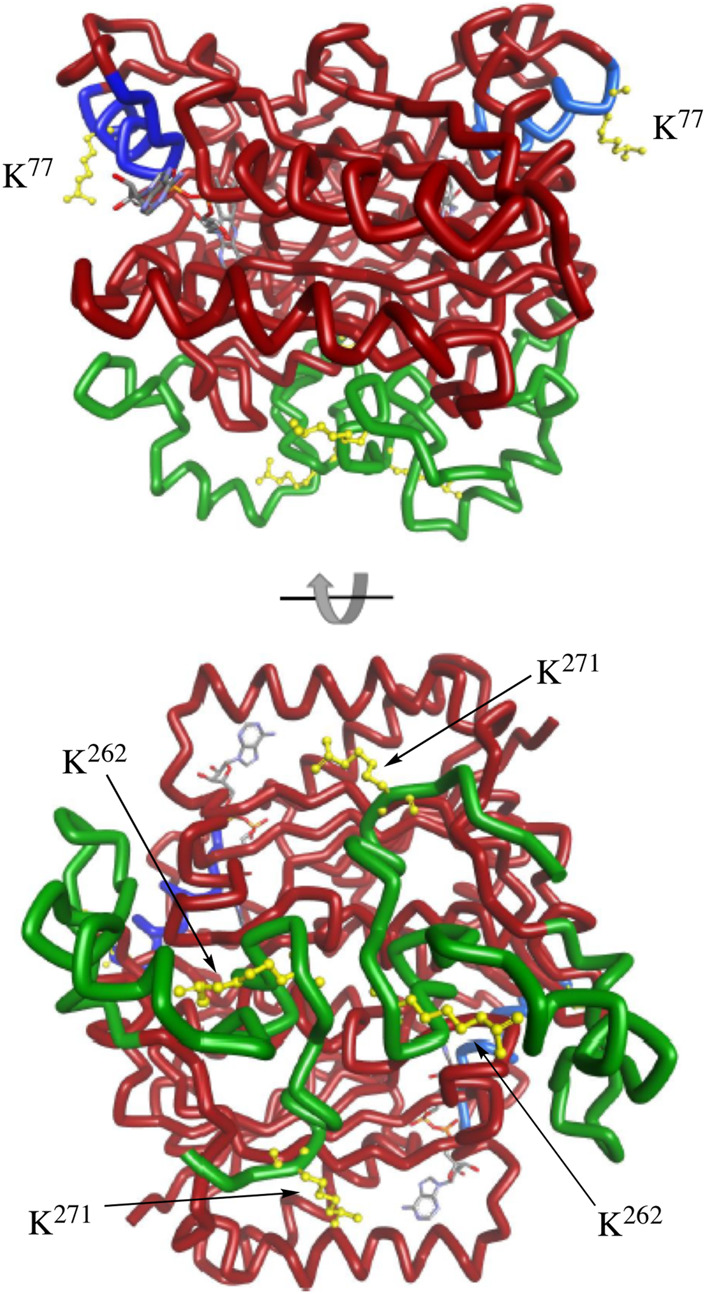
Molecular modeling of NQO1. The crystal structure of human NQO1 (1d4a) was uploaded to BIOVIA Discovery Studio 2018 for protein visualization. The homodimer of NQO1 is shown with FAD molecules in the binding pockets and the redox-dependent regions colored (helix seven in blue, C-terminus in green). Critical acetylated lysine residues in helix 7 (K^77^) and the C-terminus (K^262^, K^271^) are shown in yellow.

The C-terminal region of NQO1 has been studied in detail. Early studies showed that NADPH protected the C-terminal regions of purified NQO1 from tryptic digestion (Chen et al., 1994). More recent studies suggest that increased flexibility of the C-terminus may be a signal for degradation (Medina-Carmona et al., 2016; Medina-Carmona et al., 2017). Less information is known about helix seven other than this region is located on the external surface of NQO1 and does not directly participate in binding the FAD cofactor, NADPH or substrates (Faig et al., 2000). Interestingly, the MS data demonstrated that K^262^ and K^271^ on the C-terminal region and K^77^ on helix seven consistently showed the greatest magnitude change in acetylation/deacetylation revealing the critical nature of these lysine residues. Further supporting their importance, Uniprot sequence alignment reveals that K^77^, K^262^, and K^271^ are conserved across mouse, rat, pig, sheep, horse, large flying fox, lion, and human, among others. Molecular modeling of homodimeric NQO1 shows the position of the helix-7 regions relative to the C-terminal domains (Figure 8). The models also show the positions of these critical acetylated lysine residues in the C-terminal and helix seven regions (Figure 8). It is possible that the wide separation of the C-terminal domains and helix-7 on the external surface of NQO1 (Figure 8) allows these regions to play a role in redox-dependent protein-protein interactions.

The acetylation landscape of a protein is the result of a balance between acetylation (acetyltransferase enzymes and non-enzymatic acetylation by Ac-CoA and Ac-GSH) and deacetylation catalyzed by deacetylases including HDACs and the sirtuins. In this study we have shown that NQO1 is readily deacetylated by multiple isoforms (1-3) of the NAD^+^-dependent sirtuin family. While this is the first report of the sirtuins catalyzing the deacetylation of NQO1, immunoprecipitation studies have identified NQO1 bound to SIRT1 (Tsvetkov et al., 2021) and SIRT2 (Kang et al., 2018). In addition, we have previously shown that NQO1 co-localized with SIRT2 near the centrosome in human cells (Siegel et al., 2018) and both NQO1 and SIRT2 modulate the acetylation of α-tubulin in microtubules in peri-nuclear regions of the cell (Skoge and Ziegler, 2016; Siegel et al., 2021). Models have been proposed where NQO1 could provide NAD^+^ for sirtuin-dependent deacetylation of other target proteins (Kim et al., 2014; Kang et al., 2018; Khadka et al., 2018; Qiu et al., 2022), or alternatively, sirtuin enzymes may modulate the redox-conformation of NQO1 through the consumption of NAD^+^ ultimately lowering NADH levels (Siegel et al., 2018). The direct deacetylation of NQO1 by sirtuins raises the possibility that NQO1 may assist in its own deacetylation by providing NAD^+^ for sirtuin activity. The ability of multiple sirtuin isoforms to catalyze the deacetylation of NQO1 is not surprising since it is believed that sirtuin target protein selectivity may be achieved through the sequestration of individual sirtuin isoforms to select organelles through the use of localization domains (Bheda et al., 2016; Kupis et al., 2016).

NQO1 has been observed in the cytoplasm (Lind et al., 1990; Winski et al., 2002), nucleus (Winski et al., 2002) and mitochondria (Beyer et al., 1997) and (Supplementary Figure S4) in human and animal cells which supports deacetylation of NQO1 by multiple sirtuin isoforms. Mitochondrial localization of NQO1 is intriguing since mitochondria can also support an environment to promote the non-enzymatic acetylation of proteins by acetyl donors such as Ac-CoA and Ac-GSH (Wagner and Payne, 2013; James et al., 2017). Acetyl-donors such as Ac-CoA and Ac-GSH can transmit information about the metabolic state of the cell to protein targets through the non-enzymatic acetylation of lysine residues and can redirect cellular processes to maintain metabolic homeostasis (James et al., 2018). Under basal physiologic circumstances, sirtuin deacetylases strike a balance in lysine acetylation occupancy among protein pathways that allows for a dynamic and rapid response to changes in metabolism. The compartmentalization of pyridine nucleotide cofactors (NAD^+^:NADH and NADP^+^:NADPH) is also a unique cellular feature that is tightly regulated to direct biochemical reactions, including those catalyzed by oxidoreductases in response to cellular stress (Canto et al., 2015; Cambronne and Kraus, 2020). As a result of redox-dependent changes in structure and acetylation, NQO1 is well- positioned to play a role bridging metabolic and cellular stress.

These studies with purified NQO1 have identified a subset of lysine residues concentrated in or near the C-terminal or helix seven regions that undergo redox-dependent acetylation. These same lysine residues have been identified as targets of acetylation in biological samples and are well positioned to participate in protein-protein interactions. Our work has also shown that this subset of lysine residues in the C-terminal and helix seven regions can readily be deacetylated by the sirtuins and particularly, SIRT2. The combination of physical interaction, cellular colocalization, the ability of the sirtuins to deacetylate NQO1 and the ability of NQO1 to generate NAD^+^ an essential cofactor for sirtuin activity, suggests that these two proteins may have a more intimate relationship than previously imagined. Critically, results from these studies provide evidence that NQO1 may function as a key regulatory node at the crossroads of cellular redox (NADPH) and metabolism (Ac-CoA/Ac-GSH/Sirt2).

## Data Availability

The raw data supporting the conclusions of this article will be made available by the authors, without undue reservation.
