# Supplementary material for: Redox state and the sirtuin deacetylases are major factors that regulate the acetylation status of the stress protein NQO1
Source: Front Pharmacol. 2022 Nov 2;13:1015642. doi: 10.3389/fphar.2022.1015642 (PMC9667027; doi:10.3389/fphar.2022.1015642)
Supplement: Supplementary file 1 [file DataSheet1.pdf]

Supplementary material

Methods for Mass Spectrometry

In-gel digested peptides were loaded onto a 2cm PepMap 100 nanoviper trapping column and chromatographically resolved online with a 0.075 × 250 mm, 2.0μm Acclaim PepMap RSLC reversed-phase nanocolumn (Thermo Scientific) using a 1290 Infinity II LC system equipped with a nanoadapter (Agilent). Mobile phases consisted of water + 0.1% formic acid (A) and 90% aqueous acetonitrile + 0.1% formic acid (B). Samples were loaded onto the trapping column at 3.0 μL/min for 3.0 min under initial conditions before being chromatographically separated at an effective flow rate of 330 nL/min using a gradient of 3–8% B over 3 min and 8–40% B over 22 min at 40°C. The gradient was followed by a column wash at 75% B for 5 min. Data was collected on a 6550 Q-TOF equipped with a nano source (Agilent Technologies) operated using intensity-dependent CID MS/MS to generate peptide identifications and operated in MS-only mode for quantitation. MS/MS data was collected in positive ion polarity over a mass range of 260–1700 m/z at a scan rate of 8 spectra/second for MS scans, and a mass range of 50–1700 m/z at a scan rate of 3 spectra/second for MS/MS scans. All charge states, except singly charged species, were allowed during MS/MS acquisition. MS-Only data was collected in positive ion polarity over a mass range of 260–1700 m/z at a scan rate of 1.5 spectra/second.

Tandem mass spectra were extracted, searched, and summarized using SpectrumMill v.6 software (Agilent). The spectra were searched against the UniprotKB SwissProt [2022_02] Homo sapiens database using trypsin and allowing up to 4 missed cleavages with fixed carbamidomethyl (C), deamidation (NQ), oxidation (M), and acetylation (K) modifications. The monoisotopic peptide mass tolerance allowed was ± 20.0 ppm, and the MS/MS tolerance was ± 50.0 ppm. A false discovery rate (FDR) filter of 1% was used and a peptide score cut-off of 8 and

scored peak intensity of 50% were used to filter out poorly annotated MS/MS spectra. An AMRT library was generated from all identified NQO1 peptides.

Data files collected in MS-only mode were extracted and aligned using Profinder V.B.10.01 software (Agilent Technologies). The NQO1 AMRT library generated from MS/MS data was used to perform a batch-targeted feature extraction. Data were extracted with an ion count threshold set to two or more ions, 12000 counts, and a score threshold of 70. The scoring algorithm takes into account mass accuracy, isotope abundances, and isotope spacing of compounds based on the chemical formula of the target peptides within a specified retention time window. Data was extracted allowing charge states 1–6 with H^+ charge carriers using the peptide isotope model. The retention time window and mass window alignment tolerances were set to 0.3 min and 10 ppm, respectively. Extracted peptide peak areas were then exported to mass profiler professional v. 15.1 software (MPP) (Agilent) for statistical analysis.

To ensure that acetyl peptide data was a reflection of actual acetylation levels and not just total NQO1 protein levels, an external scalar normalization was applied based on the total NQO1 protein signal within each sample. Non-acetylated NQO1 peptide areas were summed to determine the total amount of NQO1 protein signal within each sample. The total NQO1 area sum of each sample was divided by the total area sum of the lowest sample to yield a correction factor that could be used as an external scalar normalization value for the acetyl peptide data.

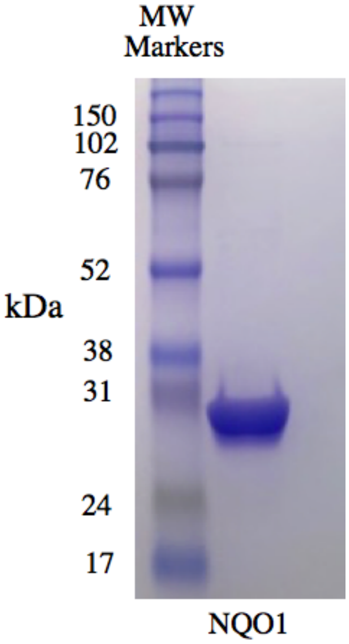

Fig S1. Purity of NQO1 was determined by SDS-PAGE. Recombinant human NQO1 (10µg, NQO1/DT-diaphorase, Millipore-Sigma #D1315) was analyzed for purity using SDS-PAGE with Coomassie blue staining (Imperial protein stain, Thermo-Fisher). The purity of NQO1 was estimated to be greater than 98%.

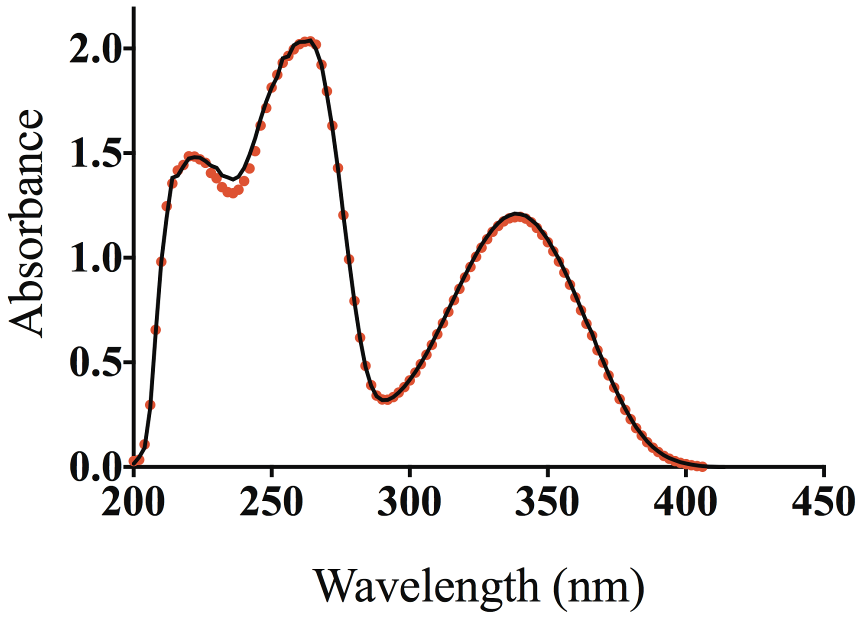

Fig S2. UV/Vis spectral analysis of NADH (200µM in 200mM potassium phosphate buffer, pH 7.4) before the addition of acetic anhydride (black line) and 5min after the addition of 125µM acetic anhydride (red dots). Experiments were performed in a 1ml quartz cuvette at 25ºC and analyzed using a HP8452 diode array spectrophotometer.

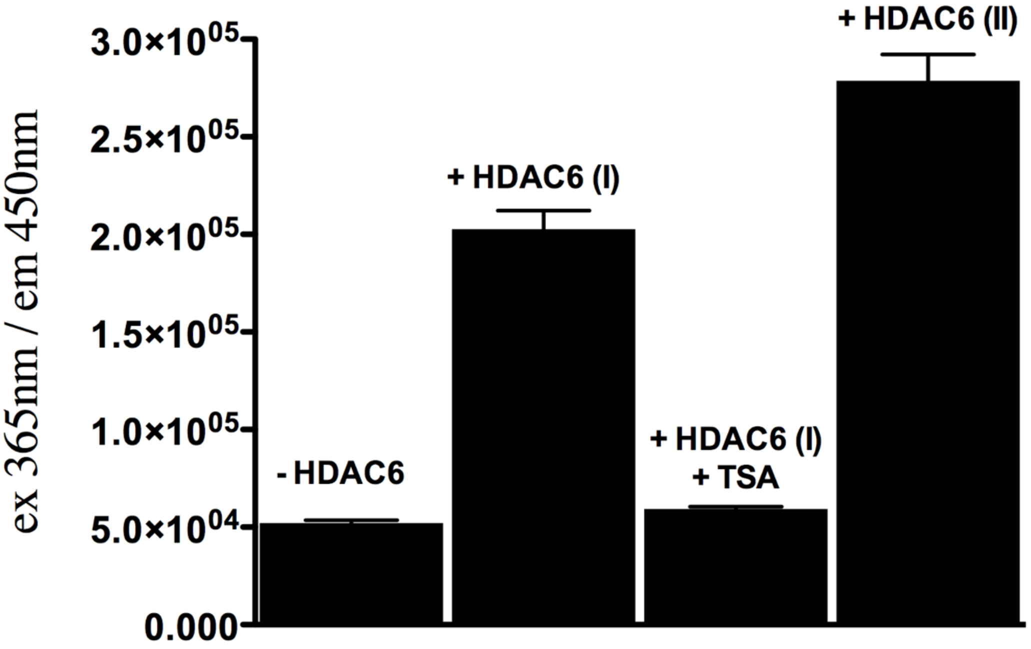

Fig S3. HDAC6 catalytic activity assay. Purified HDAC6 used in Figure 6 was assayed for catalytic activity using the substrate Fluor De Lys-SIRT1-($p53^{379-382}$)/developer as described by the manufacturer (HDAC6 Fluorometric Drug Discovery Kit, #BML-AK516, Enzo Life Sciences, Farmingdale, NY). Briefly, HDAC6 (500ng) was incubated with 15µM substrate in assay buffer (50mM Tris HCl, pH 8.0, 137mM NaCl, 2.7mM KCl, 1mM $MgCl_2$) at 37ºC in the absence and presence of trichostatin A (TSA, 500nM). After 30min, an equal volume (50µl) of Fluor De Lys developer/TSA was added to each sample and the fluorescence (excitation 365nm / emission 450nm) was measured after 5min using a Biotek Cytation 3 microplate reader. HDAC6 (I), purified recombinant human HDAC6 used in Figure 6 (3 freeze thaws); HDAC6 (II), new purified recombinant human HDAC6 from kit (1 freeze thaw). Data (n=4) is expressed as mean ± standard deviation.

3T3L1 Fibroblasts

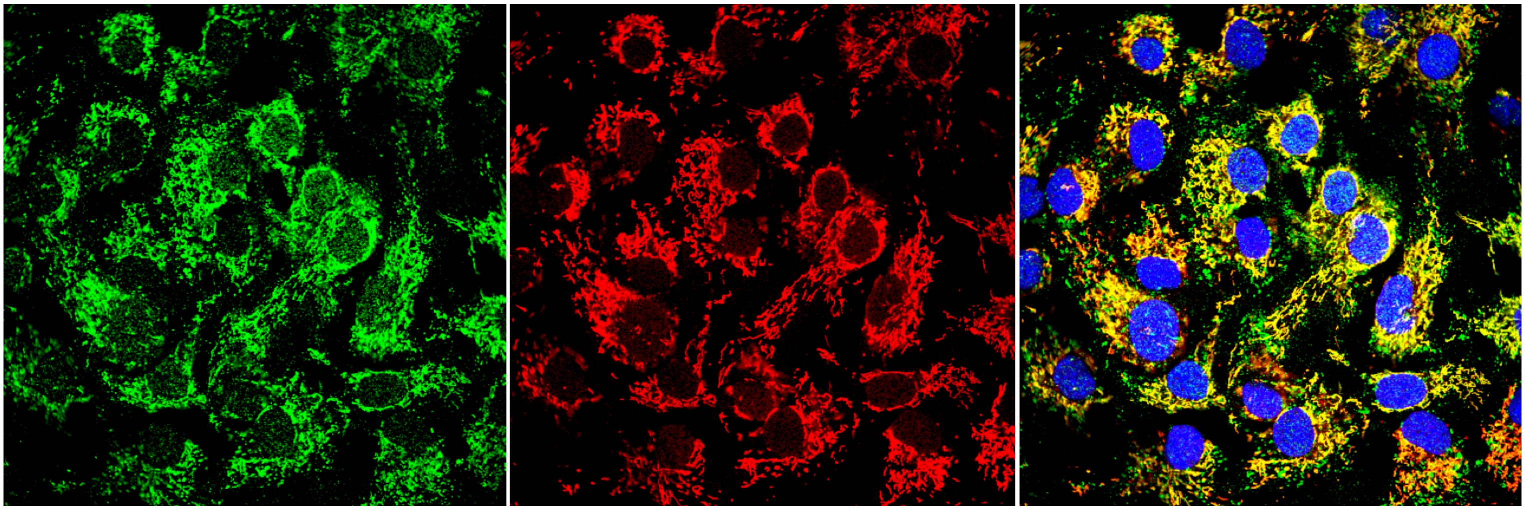

NQO1 MitoTracker Red merged/DAPI

Fig S4. Co-localization of NQO1 with mitochondria. Confocal images of mouse 3T3L1 cells showing immunostaining for NQO1 overlaid with mitochondrial staining with MitoTracker Red. Cells were treated with 100nM Mitotracker Red (ThermoFisher) for 15min at 37ºC then fixed and permeabilized as described (Siegel et al., 2018). Samples were immunostained for NQO1 using a rabbit anti-NQO1 antibody (Abcam cat #Ab34173) at a 1:1000 dilution overnight at 4ºC.

Siegel, D., Dehn, D.D., Bokatzian, S.S., Quinn, K., Backos, D.S., Di Francesco, A., et al. (2018). Redox modulation of NQO1. *PLoS One* 13(1), e0190717. doi: 10.1371.
